# Hospital clinical pathways for children affected by juvenile idiopathic arthritis

**DOI:** 10.1186/s13052-018-0576-8

**Published:** 2018-11-20

**Authors:** L. Cavazzana, M. Fornili, G. Filocamo, C. Agostoni, F. Auxilia, S. Castaldi

**Affiliations:** 1 Post Graduate School of Public Health, University of Milan, Milan, Italy; 2 Department of Clinical Sciences and Community Health, University of Milan, Milan, Italy; 3 Intermediate Pediatric Care Unit, Fondazione IRCCS Ca’ Granda Ospedale Maggiore Policlinico, Milan, Italy; 4 Department of Biomedical Sciences for Health, University of Milan, Milan, Italy; 5 Fondazione IRCCS Ca’ Granda Ospedale Maggiore Policlinico, Milan, Italy; 6 Quality Unit, Fondazione IRCCS Ca’ Granda Ospedale Maggiore Policlinico, Milan, Italy

**Keywords:** Juvenile idiopathic arthritis (JIA), Diagnostic therapeutic assistance pathway (DTAP), Quality of life, Pediatrics

## Abstract

**Background:**

Juvenile idiopathic arthritis (JIA) is the most common pediatric chronic rheumatic disease, which requires constant follow-up over the years, due to relapses during its progression. To maintain a good quality of life, it is important to limit admissions as far as possible. With the development of a Diagnostic Therapeutic Assistance Pathway (DTAP), we aim to select patients with suitable clinical conditions to be moved from routine hospital management to day care or outpatient treatment, evaluating the number of patients to whom this would apply.

**Methods:**

Monocentric study regarding admissions for JIA between 2014 and 2016 in a Pediatric Unit of a university hospital in Milan. Through an analysis of the medical records, relevant information was extracted and collected in a Microsoft™ Excel database; starting from the data collected during the first year, a DTAP was prepared for patients with active arthritis and appropriate clinical conditions.

**Results:**

The study includes data from 223 JIA hospitalization cases involving 127 patients. Applying DTAP criteria, 32% patients would have avoided admissions and 23% would have been admitted less frequently. The data concerning the activities of the Unit for JIA patients showed a relevant drop in the number of hospitalizations since 2015, from 89 in 2014 to 66 and 68 in 2015 and 2016 respectively.

**Conclusion:**

The opportunity offered by DTAP, has suggested feasible changes in hospitalization management and it’s use would promote the possibility of treating the children without hospitalization, or minimizing it. In conclusion DTAP application is a priority for the continuous improvement of clinical practice and quality of life for patients and their families.

## Background

Juvenile idiopathic arthritis (JIA) is the most common chronic rheumatic disease (prevalence of 1: 1000) in the pediatric field, which requires constant follow-up over the years since its progression is characterized by relapses [1–3]. Currently, there are no identified drugs to cure the disease, but it is possible to keep it under control and avoid the occurrence of injuries [4, 5].

In general, the management of a JIA patient is based on a multi-professional approach which, in addition to the pediatrician, also involves the nurse, the physiotherapist and the occupational therapist [6].

At the onset, the disease is frequently treated with non-steroidal anti-inflammatory drugs and intra-articular corticosteroid injections [7, 8], followed by second-level drugs such as Methotrexate or Sulfasalazine [9, 10]. In the event that a patient with JIA, in therapy with these drugs, manifests an unsatisfactory control of the disease, it is recommended to initiate treatment with biological drugs [11, 12], nowadays considered as effective and safe in treating the disease [13, 14]. The widespread use of biological drugs is one of the causes of the increase in health care costs for people with JIA in recent years; moreover, according to a European study published in 2016, also a longer length of admissions contributes to the increased spending [15].

Studies show that JIA involves a noticeable reduction in quality of life in over half of affected patients [16–18]; it therefore seems desirable to try to construct a care flowchart to provide the necessary therapies, and keeping, as far as possible, the usual level of quality of life. For chronic pediatric diseases, JIA in particular, the implementation of the Chronic Care Model is shown to be related to an improvement in the control of the disease, the management of symptoms and quality of life. This model aims at a proactive management of the pathology, and promote, among others, the use of standardized pathways, the coordination of care and greater involvement and self-management of the patient and the caregiver [19, 20].

In this sense we can hypothesize building a pathway which reserves ordinary hospitalization only for the most severe patients and privileges the outpatient or day care regimen.

The formulation of a specific Diagnostic Therapeutic Assistance pathway (DTAP) could be a useful tool to define patients who could actually avoid hospitalization, without health risks and who would benefit in terms of psycho-physical wellbeing.

The DTAPs constitute set of services provided in order to address the patient’s needs from the time of diagnosis to recovery; they are aimed at managing a specific pathology in a specific group of patients within a well-defined local context [21]. The paths are identified in a multidisciplinary manner to include all the activities carried out by the different professionals involved to manage the pathology in question. Depending on the illness for which the DTAP is implemented, activities may be planned within a single hospital organizational structure, or a much wider range of services may be involved, different in terms of reference discipline and the setting where they are supplied [22].

A review of literature published in June 2009 [23] shows that DTAPs may represent an effective mechanism to promote adherence to treatment guidelines and protocols as they reduce the variability of clinical practice. The study also found that, for predictable care pathways, DTAPs can be effective in promoting proactive disease management and ensuring that patients receive timely intervention and clinical evaluations. Furthermore, the use of these pathways leads to greater agreement among clinicians on treatment options and provides valuable support for decision-making.

The present work therefore aims at drawing up a specific DTAP for children affected by JIA with the aim of transferring patients with well defined clinical conditions from management with regular hospitalization to day or outpatient regimes. Moreover, we also wanted to assess whether this pathway is sustainable for a substantial proportion of patients.

The DTAP is outlined based on the clinical data of patients admitted to the Intermediate Pediatric Care Unit, Fondazione IRCCS Ca′ Granda, Ospedale Maggiore Policlinico of Milan.

## Methods

The analysis is based on data from a monocentric study regarding admissions for JIA in the 2014–2016 three-year period at the Intermediate Pediatric Care Unit, Fondazione IRCCS Ca′ Granda, Ospedale Maggiore Policlinico of Milan.

The study examined all admissions in the period from 1 January 2014 to 31 December 2016 where the cause of hospitalization was closely linked to JIA. A total of 223 files were assessed, 89 in 2014, 66 in 2015, and 68 in 2016, respectively. At the end the final population was made up by 127 patients, given the presence of numerous multiple admissions during the period considered.

Although the study is centered on a pediatric setting, adults of up to 23 years were also included since patients continued to be monitored also after the age of 16.

The relevant information was extracted from the printed medical records and collected in a Microsoft™ Excel database.

Socio-demographic variables (name, surname, nosographic code, date of birth, sex and municipality of residence), clinical variables of interest (diagnosis in letter of discharge, associated diseases, general conditions), presence of indicative clinical signs of systemic compromise (fever upon admission) were all included in the database. Data regarding the access modalities to the ward (planned or urgent hospitalization, transfers from other wards), variables concerning hospitalization (date of admission and discharge, reason for admission, diagnostic investigations, specialist and physiotherapy visits), the possible execution of surgery in the operating room in sedation (arthrocentesis intervention) and intravenous therapy have been recorded too.

**Fig. 1:**
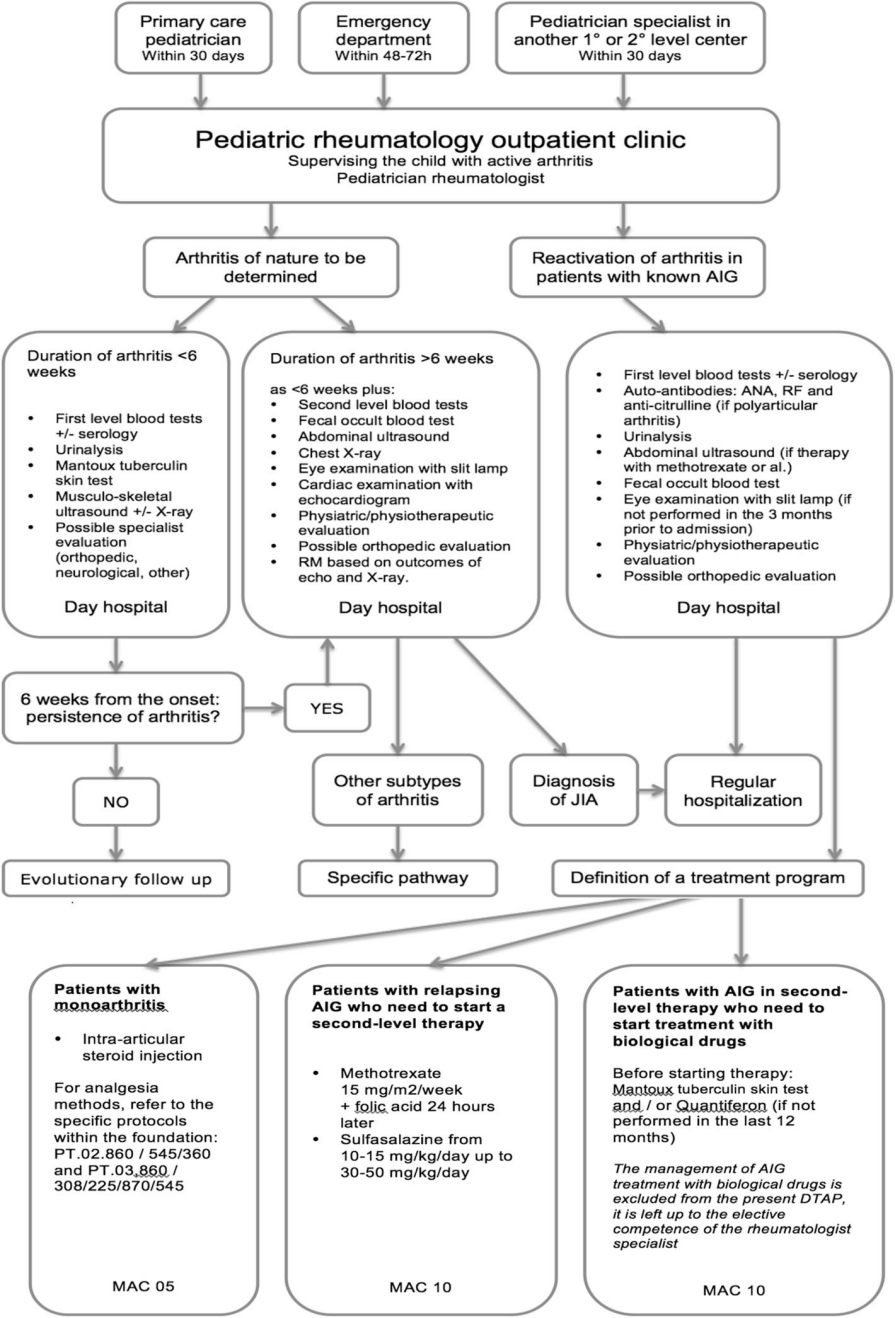
DTAP flowchart

Starting from the data collected during the first year of the study, a DTAP was set up to treat and follow children shifting from the regimen, provided by a regular hospitalization, to a day service model of care Fig. 1.

In particular, the Complex Outpatient Macroactivity with High Resource Integration (MAC) was identified as an organizational model set up by the Lombardy Region dealing with the management of all the activities that, although having to be carried out in a hospital context, can be performed in a complex outpatient procedure.

Elaboration and evaluation of the data lead to select the following DATP inclusion criteria: active arthritis, good general clinical conditions, no fever, no symptoms or signs of systemic involvement, no need of intravenous therapy or sedation. Consequently, this pathway can’t apply to patients with systemic JIA.

The only exception involved admissions due to the administration of biological therapy. It was assessed that these could be included in the DATP, as the intravenous therapy carried out on these occasions was planned and not linked to the present state of the disease. Furthermore, exclusively in this case, a day service model has been considerate adequate for children with systemic JIA, as these admissions didn’t concern a state of onset or flare of the pathology.

The DTAP was then applied to all the patients’ admissions examined in order to assess what effects its implementation could have in the clinical reality investigated.

### Statistical analysis

To visualize associations among variables multiple correspondence analysis has been used [24]. This technique represents categorical variables into a space with dimensions ordered by the proportion of the total variability that they explain. The distance between points in this space is a measure of the dissimilarity between the corresponding categories. If the original variables are strongly correlated, the first two axes explain a great proportion of the overall variability, so that the relationships among categories are well represented in that plane. DTAP was added as a supplementary variable to the space determined by the other variables.

The association between inclusion in the pathway and other variables was investigated by Firth logistic regression models [25]. Firth’s approach was adopted to obtain finite odds ratios estimates also in the case of quasi-complete separation. From the regression models 95% confidence intervals and *p*-values were also obtained. The analyses were performed with *R* [26] version 3.4.2 and its add-on packages *ca*, *FactoMineR* and *logistf*.

## Results

Demographic features of the 127 patients are summarized in Table 1: 80% of young patients are female, with a median age of 9 years; 87% of children are resident in Lombardy region. Applying DTAP criteria, in the three years period 32% of patients could have avoided hospitalization completely, and another 23% would have been hospitalized fewer times.

**Table 1:** Characteristics of the patients

Variable	
Age, years, median (IQR)	9 (5–13)
Gender, *n* (%)	
Female	102 (80)
Male	25 (20)
Residence, *n* (%)	
Milan metropolitan area	66 (52)
Rest of Lombardy	44 (35)
Other regions	17 (13)
Patients whose admissions are eligible for DTAP, *n* (%)	
No admission	57 (45)
Some admissions	29 (23)
All admissions	41 (32)

*DTAP* diagnostic therapeutic assistance pathway, *IQR* interquartile range

Inclusion in the DTAP is positively associated with residence outside Lombardy (OR = 4.12, 95% CI = 1.74–10.7), x-rays (OR = 2.04, 95% CI = 1.19–3.56) and orthopedic examination (OR = 2.78, 95% CI = 1.29–6.26).

Inclusion in the DTAP was negatively associated with urgent admission (OR = 0.20, 95% CI = 0.06–0.66), electrocardiogram (OR = 0.39, 95% CI = 0.21–0.71) and anesthesiology examination (OR = 0.04, 95% CI = 0.02–0.09). Furthermore, DTAP is associated with the cause of the hospitalization, with the odds of inclusion in the DTAP being smaller for arthrocentesis (OR = 0.07, 95% CI = 0.01–0.22) and greater for biologic therapy 61.4 (95% CI = 8.03–7894) with respect to flare Table 2.

**Table 2:** Results of univariable Firth’s logistic regression: inclusion in the DTAP versus patients and hospitalization characteristics

Variable	All (*n* = 223)	Non DTAP (*n* = 131)	DTAP (*n* = 92)	OR (95% CI)	*P*
Condition on admission, *n* (%)					
Not good	40 (18)	40 (31)	0 (0)		
Good	183 (82)	91 (69)	92 (100)		
Fever on admission, n (%)					
No	207 (88)	115 (88)	92 (100)		
Yes	16 (7)	16 (12)	0 (0)		
Sedation, *n* (%)					
No	133 (60)	41 (31)	92 (100)		
Yes	90 (40)	90 (69)	0 (0)		
Age, years, median (IQR)	10 (6–14)	10 (5–14)	10 (6–13)	1.01 (0.96–1.06)	0.70
Gender, *n* (%)					0.10
Male	54 (24)	37 (28)	17 (18)	reference	
Female	169 (76)	94 (72)	75 (82)	1.71 (0.91–3.32)	
Residence, *n* (%)					0.001
Lombardy	198 (89)	124 (95)	74 (80)	reference	
Other regions	25 (11)	7 (5)	18 (20)	4.12 (1.74–10.7)	
Discharge JIA diagnosis, *n* (%)					0.12*
Oligoarticular	84 (38)	47 (37)	37 (41)	reference	
Polyarticular	76 (35)	43 (34)	33 (36)	0.98 (0.52–1.82)	0.94
Systemic	33 (15)	26 (20)	7 (8)	0.36 (0.14–0.86)	0.02
Enthesitis-related	9 (4)	5 (4)	4 (4)	1.04 (0.26–3.91)	0.96
Monoarticular	9 (4)	3 (2)	6 (7)	2.35 (0.62–10.4)	0.21
Psoriatic	8 (4)	4 (3)	4 (4)	1.27 (0.31–5.22)	0.74
Unknown	4	3	1		
Urgent admission, *n* (%)					0.008
No	201 (90)	112 (85)	89 (97)	reference	
Yes	22 (10)	19 (15)	3 (3)	0.20 (0.06–0.66)	
Transfer from other hospital, *n* (%)					0.34
No	218 (98)	127 (97)	91 (99)	reference	
Yes	5 (2)	4 (3)	1 (1)	0.35 (0.04–3.06)	
First hospitalization, *n* (%)					0.37
No	196 (88)	113 (86)	83 (90)	reference	
Yes	27 (12)	18 (14)	9 (10)	0.68 (0.29–1.58)	
Length of stay, days, median (IQR)	6 (3–8)	6 (3–9)	6 (3–7)	0.95 (0.89–1.01)	0.11
Cause of hospitalization, *n* (%)					< 0.0001*
Flare	79 (35)	44 (34)	35 (38)	reference	
Arthrocentesis	47 (21)	45 (34)	2 (2)	0.07 (0.01–0.22)	< 0.0001
Investigations for suspected JIA	45 (20)	28 (21)	17 (18)	0.77 (0.36–1.61)	0.49
Clinical and therapeutic re-evaluation	16 (7)	6 (5)	10 (11)	2.02 (0.70–6.22)	0.19
Associated conditions	10 (4)	6 (5)	4 (4)	0.87 (0.22–3.09)	0.83
Biologic therapy	24 (11)	0 (0)	24 (26)	61.4 (8.03–7894)	< 0.0001
Arthrocentesis and biological therapy	2 (1)	2 (2)	0 (0)	0.25 (0.00–3.21)	0.32
Electrocardiogram, *n* (%)					0.002
No	58 (26)	24 (18)	34 (37)	reference	
Yes	164 (74)	106 (82)	58 (63)	0.39 (0.21–0.71)	
Unknown	1	1	0		
Ultrasound, *n* (%)					0.08
No	65 (29)	44 (34)	21 (23)	reference	
Yes	158 (71)	87 (66)	71 (77)	1.69 (0.93–3.13)	
X-rays, *n* (%)					0.01
No	98 (44)	67 (51)	31 (34)	reference	
Yes	125 (56)	64 (49)	61 (66)	2.04 (1.19–3.56)	
Nuclear magnetic resonance, *n* (%)					0.66
No	191 (86)	111 (85)	80 (87)	reference	
Yes	32 (14)	20 (15)	12 (13)	0.84 (0.39–1.79)	
Computed tomography, *n* (%)					0.69
No	219 (98)	129 (98)	90 (98)	reference	
Yes	4 (2)	2 (2)	2 (2)	1.43 (0.22–9.42)	
Positron emission tomography, *n* (%)					0.20
No	220 (99)	128 (98)	92 (100)	reference	
Yes	3 (1)	3 (2)	0 (0)	0.20 (0.00–2.08)	
Eye examination, *n* (%)					0.09
No	100 (45)	65 (50)	35 (38)	reference	
Yes	123 (55)	66 (50)	57 (62)	1.60 (0.93–2.75)	
Cardiac examination, *n* (%)					0.19
No	101 (45)	64 (49)	37 (40)	reference	
Yes	121 (55)	66 (51)	55 (60)	1.44 (0.84–2.47)	
Unknown	1	1	0		
Anesthesiology examination, *n* (%)					< 0.0001
No	127 (57)	42 (32)	85 (92)	reference	
Yes	96 (43)	89 (68)	7 (8)	0.04 (0.02–0.09)	
Orthognatodontic examination, *n* (%)					0.08
No	182 (82)	112 (85)	70 (76)	reference	
Yes	41 (18)	19 (15)	22 (24)	1.84 (0.94–3.65)	
Orthopedic examination, *n* (%)					0.009
No	193 (87)	120 (92)	73 (79)	reference	
Yes	30 (13)	11 (8)	19 (21)	2.78 (1.29–6.26)	
Dermatological examination, *n* (%)					0.70
No	209 (94)	122 (93)	87 (95)	reference	
Yes	14 (6)	9 (7)	5 (5)	0.81 (0.26–2.34)	
Otorhinolaryngology examination, *n* (%)					0.76
No	221 (99)	130 (99)	91 (99)	reference	
Yes	2 (1)	1 (1)	1 (1)	1.43 (0.11–17.8)	
Physical therapy, *n* (%)					0.96
No	70 (31)	41 (31)	29 (32)	reference	
Yes	153 (69)	90 (69)	63 (68)	0.99 (0.56–1.76)	
Arthrocentesis, *n* (%)					< 0.0001
No	114 (51)	43 (33)	71 (77)	reference	
Yes	109 (49)	88 (67)	21 (23)	0.15 (0.08–0.27)	
Intravenous therapy, *n* (%)					< 0.0001
No	104 (47)	24 (18)	80 (87)	reference	
Yes	119 (53)	107 (82)	12 (13)	0.04 (0.02–0.07)	

*DTAP* diagnostic therapeutic assistance pathway, *JIA* juvenile idiopathic arthritis, *IQR* interquartile range, *OR* odds ratio, *CI* confidence interval

^*^Global likelihood ratio test p-value

**Fig. 2:**
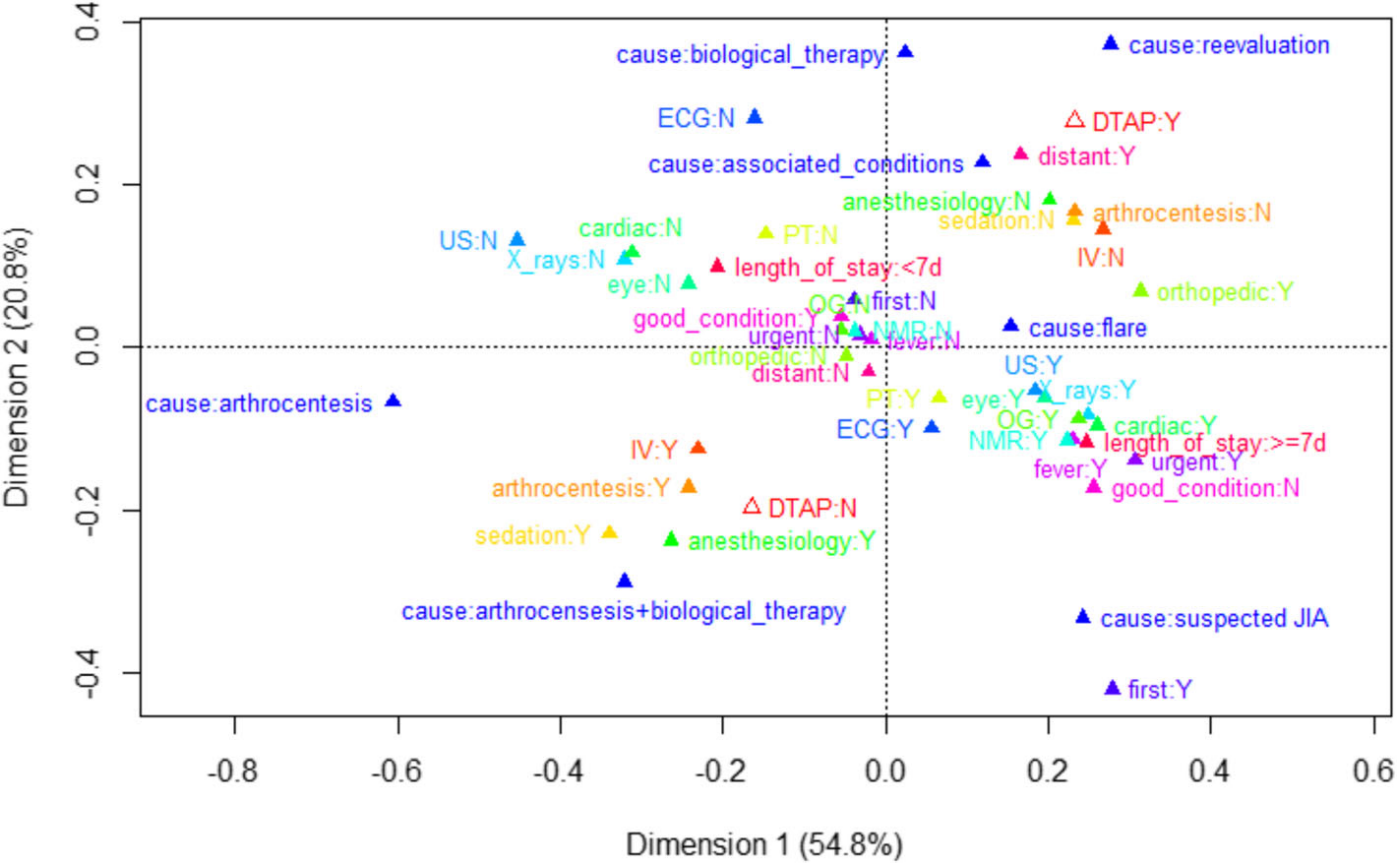
First two dimensions plane from the multiple corresponding analysis. Categorical variables: anesthesiology examination (*anesthesiology*), arthrocentesis, cardiac examination (*cardiac*), cause of hospitalization (*cause*), length of stay, electrocardiogram (*ECG*), eye examination (*eye*), fever on admission (*fever*), first hospitalization (*first*), good condition on admission (*good condition*), intravenous therapy (*IV*), nuclear magnetic resonance (*NMR*), orthognatodontic examination (*OG*), orthopedic examination (*orthopedic*), residence outside Lombardy region (*distant*), physical therapy (*PT*), sedation, ultrasound (*US*), urgent admission (*urgent*), X-rays. Eligibility to the diagnostic therapeutic care pathway (DTAP) is passively projected to the plane as a supplementary variable. The percentage of total variability explained by each axis is reported. Other abbreviations: *JIA* = juvenile idiopathic arthritis; *N* = no; *Y* = yes

In the multiple correspondence analysis, the first two axes explain 75.6% of the total variability, so that relationships among categories are well represented in this plane. The plot shows positive association among the following categories: inclusion in the DTAP, orthopedic examinations, residence outside the region and hospitalization due to either flare, re-evaluation, associated conditions or biological therapy. On the contrary, exclusion from the DTAP is associated to anesthesiology examination, sedation, arthrocentesis and intravenous therapy and arthrocentesis-related admissions Fig. 2.

Furthermore, the data concerning the activities carried out by the Intermediate Pediatric Care Unit for patients with JIA highlight how the number of admissions has been reduced from 2014 to 2016. On the contrary, outpatients’ activities like MAC have been constantly rising during the observed years Table 3.

**Table 3:** Intermediate Pediatric Care Unit’s activity data about patients with JIA

Activity/year	2014	2015	2016
First examination	152	201	165
Follow-up	524	613	585
Mac10	175	193	222
Hospitalization	89	66	68

*MAC* Complex Outpatient Macroactivity with High Resource Integration (an organizational model, set up by the Lombardy Region, relating the management of all the activities that could be done outpatient)

## Discussion

Starting from the data collected during the first year of the study, a DTAP was set up to identify patients who could be treated in regimen alternative to regular hospitalization. It was therefore a real-time process, enabling the establishment of a pathway suitable for the setting of application. In this way, we could evaluate and modify the hypothesized variables a priori to better reflect the clinical reality.

The analysis of the 223 cases of admissions of young JIA patients figured out a picture of the typology of patients attending the department and the services performed during hospitalization. Regarding the epidemiology of JIA, the data are in line with literature reports: females are more affected than males, there are children of all ages, the most represented form is oligoarticular arthritis, followed by polyarticular and systemic arthritis.

Data examination relative to patient residency showed that 52% of patients reside in the Milan metropolitan area, which would make the transition to outpatient treatment not only feasible but also beneficial, especially in terms of quality of life, for a sizeable majority of users.

Out of all the patients considered, as many as 55% could be impacted by the DTAP. This group would have completely avoided admissions or reduced their number over the three-year period.

The characteristics of the subjects and the services performed during hospitalization were examined and compared between the group of patients who could be included in the DTAP and those who would have confirmed the regular hospitalization pathway. Several variables were found to be associated with the inclusion or exclusion from the pathway. For example, residency outside the Lombardy region provide four times more odds of being included in the pathway; this could be explained by the fact that most of these patients are hospitalized for a clinical re-evaluation or for non-urgent reasons. Probably, also the limited availability of supportive accommodation outside the hospital had a role. On the other hand, urgent admissions, as were classified all the cases coming from the emergency room, entail a close correlation with exclusion from the DTAP as they require more direct assistance than the outpatient pathway. These hospitalizations, indeed, were mostly related to more severe cases of JIA at its onset or relapse and to acute infectious disease in patients with JIA. Furthermore, as to the causes of hospitalization, it can be observed that the programmed administration of biological therapy, compared with the reference category of the flare, is positively correlated with the DTAP, as this type of service is well suited to fit the characteristics of the MAC. Arthrocentesis indeed is negatively correlated, since in most cases it is carried out under sedation and therefore requires hospitalization. The type of JIA, instead, doesn’t seem to affect the inclusion or exclusion from the DTPA. In the two major represented type of JIA, oligoarticular and polyarticular, the percentage of admissions included and excluded from the DTAP is similar to the one relative to all the admissions. Regarding the hospitalizations of patients with systemic JIA, the majority result excluded from the DTAP (according to DTAP criteria); the remaining cases were related to the administration of biologic therapy, as it is the only case in which a day service it’s been considered proper for these patients.

The MAC graph concerning the first two axes are clearly representative of the structure of association between the variables with 75.6% of total variability.

The results are consistent with the criteria for exclusion from the DTAP, since sedation, arthrocentesis, anesthesia evaluation and intravenous therapy are inter-related each other and with non-inclusion in the DTAP.

While not yet officially approved by our institutional board, the way the planned DTAP for JIA patients has been developed (as result of collaboration between preventive medicine and pediatricians) may well represent a pathway to better addressing hospitalization, with powerful savings in health economics. As part of a trend promoted by national health policies and by the creation of the MAC by the Lombardy Region, the number of MAC-type services increased in the year following the start of the study, while the normal admissions for the management of patients with the appropriate characteristics dropped from 89 in 2014 to 66 in 2015, and 68 in 2016.

Moreover, while there is no evidence of return admissions, continuity of care seems to be ensured as a result of the provision of assistance through MAC, confirming the feasibility of introducing alternative solutions to hospitalization in controlled settings, and, accordingly, a measurable advantage for the quality of life of young people with JIA can be realistically predicted.

## Conclusions

The use of DTAP to manage JIA patients raises the possibility of treating the young chronic patient without hospitalization, or reducing it to the necessary. This can yield significant benefits, not only from the point of view of resource optimization, but especially towards the patients’ quality of life.

The application of the DTAP following its official approval represents a priority for the continuous improvement of the clinical practice and quality of life of patients and their families, and can be considered an objective to be pursued in a perspective of pediatric appropriateness.

In the near future, also the evaluation of the customer satisfaction of parents and children who benefit from outpatient management of the disease will be mandatory, to have a full view of the change of intervention policy.

## Abbreviations

DTAP: diagnostic therapeutic assistance pathway
JIA: juvenile idiopathic arthritis
MAC: complex outpatient macroactivity with high resource integration
